# Two Photon Fluorescence Microscopy of the Unstained Human Cochlea Reveals Organ of Corti Cytoarchitecture

**DOI:** 10.3389/fncel.2021.690953

**Published:** 2021-08-05

**Authors:** Janani S. Iyer, Richard Seist, In Seok Moon, Konstantina M. Stankovic

**Affiliations:** ^1^Program in Speech and Hearing Bioscience and Technology, Harvard Medical School, Boston, MA, United States; ^2^Eaton Peabody Laboratories and Department of Otolaryngology - Head and Neck Surgery, Massachusetts Eye and Ear, Boston, MA, United States; ^3^Wellman Center for Photomedicine, Massachusetts General Hospital, Boston, MA, United States; ^4^Department of Otolaryngology - Head and Neck Surgery, Harvard Medical School, Boston, MA, United States; ^5^Department of Otorhinolaryngology - Head and Neck Surgery, Paracelsus Medical University, Salzburg, Austria; ^6^Department of Otorhinolaryngology, Yonsei University College of Medicine, Seoul, South Korea; ^7^Department of Otolaryngology - Head and Neck Surgery, Stanford University School of Medicine, Stanford, CA, United States

**Keywords:** two photon fluorescence microscopy, human cochlea, organ of Corti, hair cells, cochlear neurons

## Abstract

Sensorineural hearing loss (SNHL) is the most common sensory deficit worldwide, and it typically originates from the cochlea. Methods to visualize intracochlear cells in living people are currently lacking, limiting not only diagnostics but also therapies for SNHL. Two-photon fluorescence microscopy (TPFM) is a high-resolution optical imaging technique. Here we demonstrate that TPFM enables visualization of sensory cells and auditory nerve fibers in an unstained, non-decalcified adult human cochlea.

## Introduction

Diagnostics and therapies for sensorineural hearing loss (SNHL) remain limited in part because of a historical lack of methods for visualizing the cochlea’s interior at the cellular level in living patients. While conventional computed tomography (CT) and magnetic resonance imaging (MRI) can reveal gross anatomical defects of the cochlea and may be sufficient for guiding otologic surgeons in planning their surgical access to the middle and inner ear regions, they do not afford the resolution necessary to enable visualization of the individual cells and auditory nerve fibers that are known from animal and human autopsy studies to be damaged in the progression of SNHL.

Recent advances in high resolution, deep penetration fluorescence microscopy techniques and the interfacing of these systems with miniature clinical endoscopes motivate investigation into whether these tools might be useful for intracochlear diagnostic applications; indeed, the abundance of endogenous fluorophores in the inner ear (e.g., flavin adenine dinucleotide (Sewell and Mroz, 1993) and nicotinamide adenine dinucleotide (Tiede et al., 2009) makes the cochlea a promising candidate for future fluorescence endoscopy. Following up on previous work in a mouse model by us (Yang et al., 2013; Romito et al., 2019) and others (Bae et al., 2020), here we demonstrate the ability of two-photon fluorescence microscopy (TPFM) to facilitate visualization of sensory cells and auditory nerve fibers in an unstained, non-decalcified cochlea from a former adult patient.

## Materials and Methods

The patient’s temporal bone was harvested at autopsy and fixed in formaldehyde according to previously described procedures (Merchant and Nadol, 1993). For TPFM imaging, the otic capsule over the cochlea’s apical, middle, and basal turns was slowly and carefully drilled away using sub-millimeter cutting and diamond burrs, revealing the organ of Corti’s surface in its entirety. The resulting specimen with exposed sensory epithelium was fixed to a glass petri dish with dental cement, submerged in phosphate buffered saline solution, and observed under a light microscope to confirm that **(a)** the structure had remained grossly intact during the drilling process, and **(b)** bone dust had been adequately flushed away. The dish was then secured to the surface of a 3-axis goniometer to allow precise tip and tilt manipulation in addition to rotation, and the entire system was then positioned under a Thorlabs Bergamo II Series Multiphoton microscope (Thorlabs, Newton, NJ, United States) for investigation using TPFM. The light source was a Spectra-Physics Mai Tai HP Ti:Sapphire laser (Spectra-Physics, Santa Clara, CA, United States) tuned to 830 or 836 nanometers and maintaining a pulse width of less than 100 femtoseconds. Emitted fluorophores were detected using Hamamatsu photomultiplier tubes (Hamamatsu Photonics, Hamamatsu City, Shizuoka, Japan). The objective lenses used were the Nikon CF175 LWD 16X and Apochromat 25XC water immersion lenses (Nikon, Minato, Tokyo, Japan).

To verify images obtained using TPFM, cochlear wholemounts were prepared for confocal immunohistochemistry. Specifically, a bone plug containing the inner ear was drilled down to the otic capsule and decalcified in EDTA. The cochlea was microdissected, and wholemounts of the organ of Corti prepared. Cochlear pieces were frozen in 30% sucrose until further use. The specimens were thawed to permeabilize and incubated in blocking buffer for 1 h. Hair cells were stained by incubating the specimen with rabbit anti-Myosin VI and VIIa (Proteus Biosciences, Ramona, CA, United States; #25-6791 and 25-6790, respectively) at 1:100 overnight at 37°C. After washing in PBS, wholemounts were incubated with a fluorescently labeled anti-rabbit secondary antibody twice for 60 min, and mounted onto glass slides in Vectashield (Vectorlabs, Burlingame, CA, United States). Images were taken on a Leica SP5 (Wetzlar, Germany) confocal microscope with a 20× glycerol objective.

This research was exempt by the Massachusetts Eye and Ear Institutional Review Board because it was based on deidentified cadaveric specimens.

## Results

**FIGURE 1:**
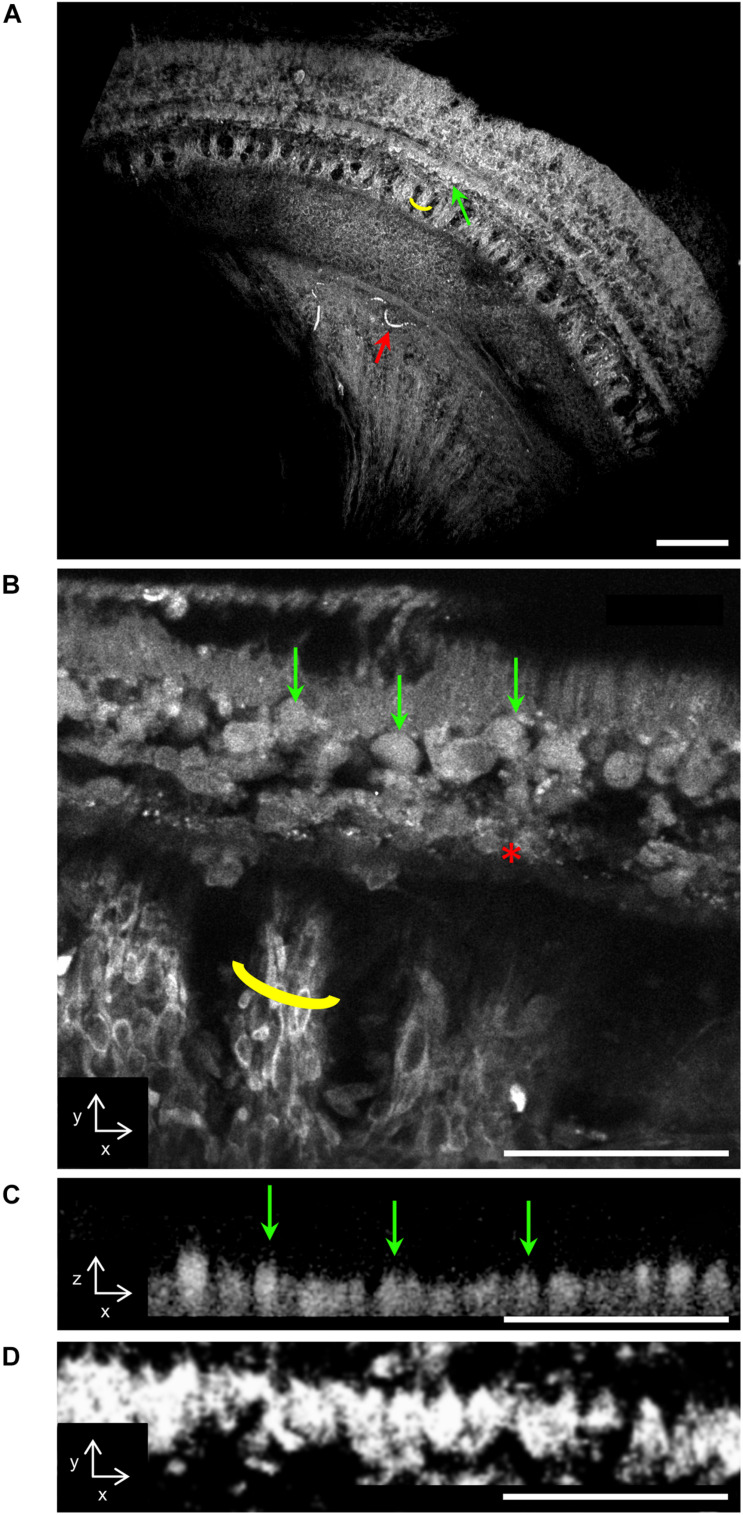
**(A)** Two-photon fluorescence microscopy (TPFM) of the unstained, *in situ* organ of Corti. Low magnification (16×) view of the basal turn of the organ of Corti. The green arrow indicates the row of inner hair cells, the yellow outline indicates auditory nerve fiber bundles, and the red arrow indicates vasculature. **(B)** TPFM high magnification (25×) view of the region depicted in panel **(A)**, revealing auditory nerve fiber bundles (yellow outline) and individual sensory (green arrows) and supporting cells (asterisk). **(C)** High magnification (25×) view of the organ of Corti’s inner hair cell (IHC) region in cross-section, revealing individual IHCs (green arrows). **(D)** Maximum projection confocal image of the organ of Corti stained with myosin VI and VIIa at the row of inner hair cells shows a similar appearance to panel C. Scale bars: 100 μm in panel **(A)**, 50 μm in panels **(B–D)**, respectively.

Despite the lack of applied fluorescent stain, a strong endogenous fluorescence signal facilitated imaging of the organ of Corti’s surface features with high levels of detail (Figure 1A). Individual sensory cells and bundles of auditory nerve fibers were clearly visualized (Figures 1A,B), in addition to winding vasculature containing small donut-shaped cells consistent with erythrocytes in regions more proximal to the modiolus (Figure 1A). The identity of the sensory cells detected with TPFM in the unstained specimen was confirmed using confocal microscopy applied to the specimen immunostained for hair cells (Figure 1D). Because access to human inner ear tissue is limited and post-mortem times are variable, and on the order of hours, preservation of human inner ear tissue is typically not as good as in animal experiments where all variables can be precisely controlled and post-mortem time is only a few minutes.

## Discussion

For over 130 years, the gold standard method for studying the cellular basis of human SNHL has been post-mortem histology (Politzer, 1892). This has required lengthy processing that typically takes many months to complete and includes decalcification, embedding, sectioning, and staining with hematoxylin and eosin—with the collection of temporal bones often being long post-mortem (Merchant and Nadol, 1993).

A scarce opportunity to collect fresh human tissue are life-threatening diseases that require the removal of the inner ear for surgical access. During these surgeries, fresh inner ear specimens can be collected and subsequently molecularly or histologically analyzed, e.g., by RNA-seq (Schrauwen et al., 2016), immunohistochemistry, transmission electron microscopy (TEM) microscopy (Liu et al., 2015), or super-resolution structured illumination microscopy (SR-SIM) (Liu et al., 2017). Another opportunity to better understand human cochlear biology is the collection of perilymph, which can be done safely during surgeries that expose the round window or labyrinth (Lysaght et al., 2011; Schmitt et al., 2017). Imaging the cochlea in living humans today relies on CT and MRI. Although significant advances have been made, clinical CT and MRI do not afford cellular or microstructural resolution (Pearl et al., 2014; van Egmond et al., 2014; Thylur et al., 2017). While synchrotron radiation phase contrast imaging (SR-PCI) could visualize the organ of Corti’s cellular architecture in intact human temporal bones *in situ* (Iyer et al., 2018), the intense radiation currently required for this type of imaging post mortem makes it unsafe for imaging in alive humans.

Here, we demonstrate that TPFM obviates the need for such laborious, lengthy, and artifact-prone processing by providing cellular and subcellular resolution of the cochlea’s interior in a non-decalcified, unstained specimen. This strongly motivates further investigation into the sources of endogenous fluorescence in the inner ear and how they might be tapped for diagnostic imaging without contrast dyes or radiation in living humans, e.g., *via* intracochlearly inserted microendoscope. In conclusion, TPFM could accelerate progress in understanding cellular-level pathology in precious human temporal bone specimens and may lead the way toward much-needed personalized therapy recommendations in living patients suffering from SNHL.

## Data Availability Statement

The raw data supporting the conclusions of this article will be made available by the authors, without undue reservation.

## Ethics Statement

Ethical review and approval was not required for the study on human participants in accordance with the local legislation and institutional requirements. Written informed consent for participation was not required for this study in accordance with the national legislation and the institutional requirements.

## Author Contributions

KS conceived and supervised the project. JI and KS designed the experiments. JI and IM performed the experiments. JI, RS, and KS analyzed the data and wrote the manuscript. All authors contributed to the article and approved the submitted version.

## Conflict of Interest

The authors declare that the research was conducted in the absence of any commercial or financial relationships that could be construed as a potential conflict of interest.

## Publisher’s Note

All claims expressed in this article are solely those of the authors and do not necessarily represent those of their affiliated organizations, or those of the publisher, the editors and the reviewers. Any product that may be evaluated in this article, or claim that may be made by its manufacturer, is not guaranteed or endorsed by the publisher.

## Supplementary Material

Supplementary Figure 1 **(A)** Experimental set-up for two-photon fluorescent microscopy of the human temporal bone. **(B)** Before imaging, the temporal bone was fixed to a petri dish with dental cement. The otic capsule was carefully drilled with an otologic drill to expose the organ of Corti. The red square indicates the area imaged in Figure 1.
