# Supplementary figures and images for: Two Photon Fluorescence Microscopy of the Unstained Human Cochlea Reveals Organ of Corti Cytoarchitecture
Source: Front Cell Neurosci. 2021 Aug 5;15:690953. doi: 10.3389/fncel.2021.690953 (PMC8376148; doi:10.3389/fncel.2021.690953)

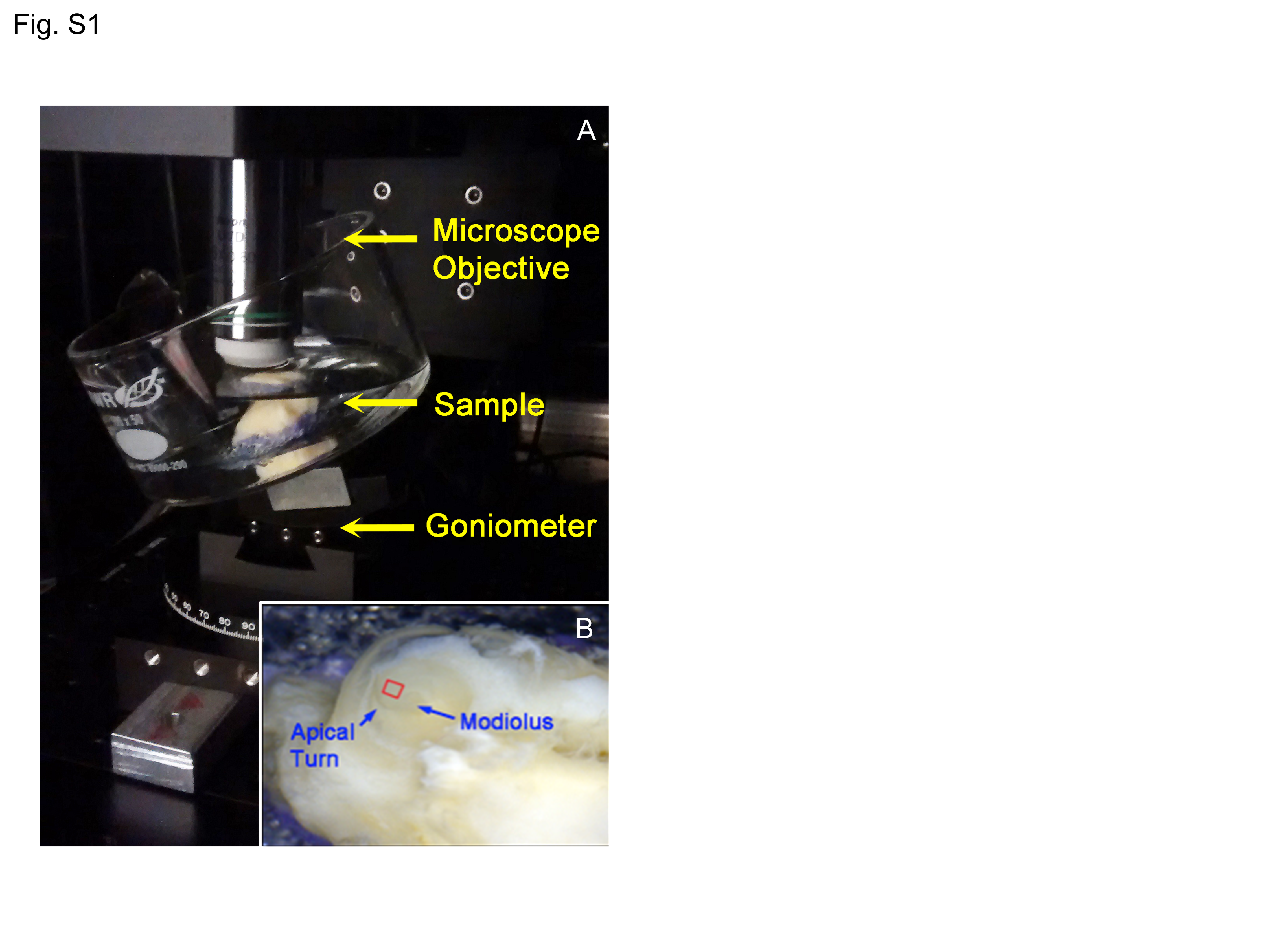

Supplement: Supplementary Figure 1 — (A) Experimental set-up for two-photon fluorescent microscopy of the human temporal bone. (B) Before imaging, the temporal bone was fixed to a petri dish with dental cement. The otic capsule was carefully drilled with an otologic drill to expose the organ of Corti. The red square indicates the area imaged in Figure 1. [file Image_1.TIF]
